# Metal Oxide Nanosheet: Synthesis Approaches and Applications in Energy Storage Devices (Batteries, Fuel Cells, and Supercapacitors)

**DOI:** 10.3390/nano13061066

**Published:** 2023-03-16

**Authors:** Arnob Das, Susmita Datta Peu, Md. Sanowar Hossain, Md Abdul Mannan Akanda, Mostafa M. Salah, Md Muzaffer Hosen Akanda, Mahbubur Rahman, Barun K. Das

**Affiliations:** 1 Department of Mechanical Engineering, Rajshahi University of Engineering and Technology, Rajshahi 6204, Bangladesh; 2 Department of Agriculture, Hajee Mohammad Danesh Science and Technology University, Dinajpur 5200, Bangladesh; 3 School of Engineering and Technology, Central Michigan University, Mount Pleasant, MI 48859, USA; 4 Electrical Engineering Department, Future University in Egypt, Cairo 11835, Egypt; 5 Department of Manufacturing Engineering, Texas State University, Waco, TX 78666, USA; 6 Ingram School of Engineering, Texas State University, San Marcos, TX 78666, USA

**Keywords:** batteries, energy storage, fuel cells, metal oxide nanosheets, molecular-scale integration, supercapacitors

## Abstract

In recent years, the increasing energy requirement and consumption necessitates further improvement in energy storage technologies to obtain high cycling stability, power and energy density, and specific capacitance. Two-dimensional metal oxide nanosheets have gained much interest due to their attractive features, such as composition, tunable structure, and large surface area which make them potential materials for energy storage applications. This review focuses on the establishment of synthesis approaches of metal oxide nanosheets (MO nanosheets) and their advancements over time, as well as their applicability in several electrochemical energy storage systems, such as fuel cells, batteries, and supercapacitors. This review provides a comprehensive comparison of different synthesis approaches of MO nanosheets, as well their suitability in several energy storage applications. Among recent improvements in energy storage systems, micro-supercapacitors, and several hybrid storage systems are rapidly emerging. MO nanosheets can be employed as electrode and catalyst material to improve the performance parameters of energy storage devices. Finally, this review outlines and discusses the prospects, future challenges, and further direction for research and applications of metal oxide nanosheets.

## 1. Introduction

In recent times, the rapid development in technologies, commercialization, and industrialization have caused a substantial increment in the requirement of global energy consumption [1,2,3]. Fossil fuels are at the top of energy sources despite having a lot of negative issues. Even, according to statistics in 2019, 84% of the total global energy was generated from fossil fuels such as natural gas, coal, and oil. Due to the use of fossil fuels for energy production, several negative impacts are occurring, such as global warming, rise in sea level, soil pollution, water pollution, and air pollution which cause significant harm to flora and fauna. Researchers are working with great concern to minimize the use of such fossil fuels for which they have carried out approaches to utilize renewable sources for the fulfillment of energy requirements. However, the development of these approaches to utilize renewable sources are not sufficient. Additionally, to utilize green sources it’s required to develop efficient energy storage devices so that it can be possible to use the energy from such storage devices whenever power and energy are required. Nowadays, molten salt nanofluids are used as thermal energy storage (TES) materials for concentrated solar power (CSP) plants for large scale power generation. However, the appropriate synthesizing of molten salt for enhanced specific heat capacity is becoming an obstacle in its application [4]. Electrochemical energy storage is the most popular way to store energy for further use and there are several systems including fuel cells, supercapacitors, and batteries which are applied to store energy thermochemically. Researchers are working towards advancements in energy storage textiles to enhance the wearability and electrochemical performances and stability of batteries, and supercapacitors for improving their applicability for several purposes [5]. Wearable energy storage devices prepared using MO nanosheets can be integrated with bioenergy from biofluids, and bioenergy from human motions, and these integrated mechanisms possess attractive potential for building self-driven body-worn electronic devices [6]. Nano structure-enhanced electrochemical energy storage devices can be integrated with self-operated sensors toward self-sustainable gadgets for the management of health and wellbeing [7].

Due to possessing a high energy density and attractive kinetics, thermochemical energy storage is the preferred system. Fuel cells possess better energy conversion potential than supercapacitors and batteries, but the limitations of fuel cells include a shorter lifespan, less durability, and high cost due to applying several expensive catalysts [8,9]. Supercapacitors possess higher cyclic stability, better energy, and power density, and better kinetics for charging and discharging than batteries, but these are limited to lower energy density and lower specific capacitance [10,11]. To enhance the lifecycle, durability, and stability of the electrode material, the phase transition reaction should have potential reversibility and there should also be a proper space where atomic-level reactions can take place [12]. Therefore, to utilize the concept of electrochemical energy storage (EES), two-dimensional (2D) metal oxide electrodes are one of the best solutions that can increase the cyclic lifetime, power, and energy density of storage devices [13,14]. Two-dimensional MO nanosheets (MO-NSs) are rapidly emerging due to their applicability in EES devices and their ability to ameliorate performance parameters. Due to the variable valence states of these 2D materials, MO-NSs possess rich redox reactions. This feature assists to improve the storage capacity of suitable electrode materials used in batteries and supercapacitors. Two-dimensional MO nanosheets possess less volume change, more active sites, and less diffusion length compared to metal oxide nanostructures. These features assist to improve the electrochemical energy storage performance by enhancing cycling stability, specific capacity, surface area, and capacitance retention. 

Two-dimensional metal oxide nanosheets possess a thickness of only a few atomic thin layers. Compared to 1D and 3D nanostructure materials, they possess several conveniences and limitations. Having a larger surface area is a major convenience of 2D nanosheets that exhibit much more reactivity than 1D and 3D nanostructures, and this feature makes them more promising for energy storage applications, such as fuel cells, supercapacitors, and batteries. These 2D nanosheets are also more suitable in sensors and flexible electronics compared to 1D and 3D nanostructures due to their flexibility feature [15,16]. However, the high cost of 2D nanosheets is the main barrier to the synthesis and fabrication of these nanosheets and these problems have limited the wide application of 2D metal oxide nanosheets in energy storage systems [17]. Overall, metal oxide (MO) nanosheets possess some uncommon attractive features that make them useful for electrochemical energy storage, but their preparation and manipulation processes may stand as a barrier during application in some cases.

**Figure 1:**
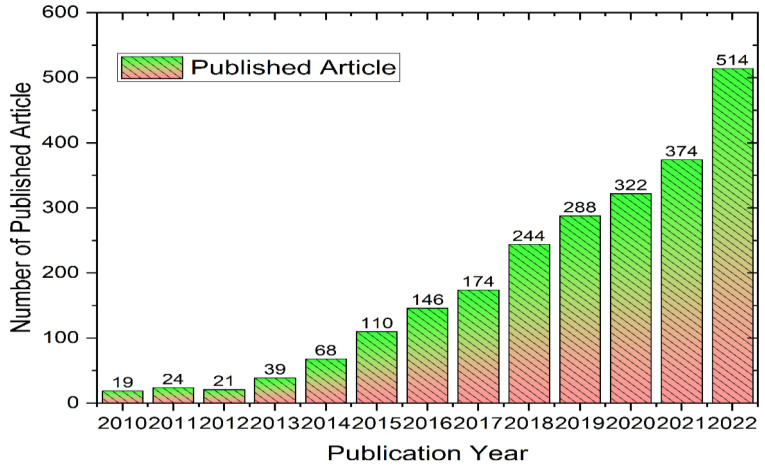
Number of publications on “metal oxide nanosheets” from 2010–2022.

Researchers have developed a lot of methodologies to synthesize 2D metal oxide nanosheets using top-down and bottom-up approaches [18,19,20,21]. Figure 1 illustrates the number of publications on metal oxide nanosheets from 2010 to 2022. The chemical vapor deposition, sol-gel method, and exfoliation with some merits and demerits are the most used approaches by researchers. In the sol-gel approach, a precursor is used to prepare a gel which is further heated to prepare nanosheets. In the case of chemical vapor deposition, the main advantages includes its capability to generate high-quality nanosheet materials with a large surface area. However, its low yield limits its wide application [22,23]. Exfoliation can also synthesize a massive amount of 2D nanosheets by applying mechanical or chemical intercalation to weaken the interlayer and molecular forces [24]. In recent years, hydrothermal and electrophoretic synthesis approaches have gained much research interest as these methodologies can provide a high yield, as well as good dimensional control [21,25,26]. 

The previously published literature reveals massive conveniences which make MO nanosheets attractive for energy storage applications including energy density, chemical stability, semi-conductivity, redox properties, flexibility, power density, and larger surface area [14,27,28]. Moreover, researchers and scientists have published a lot of articles documenting the applications of MO nanosheets as electrode material in EESs such as supercapacitors, fuel cells, and batteries [29,30,31,32]. According to the author’s knowledge, there is no literature on the several types of MO nanosheets, their synthesis protocols, and applications. Therefore, this review paper approaches to provide sufficient information about MO nanosheet advancements over time, their applicability in several EES devices, synthesis, and manipulation approaches by reviewing more than 170 papers. 

## 2. Synthesis Approaches of MO Nanosheets

### 2.1. Top-Down Synthesis

Top-down synthesis of MO nanosheets is a process of creating metal oxide nanosheets by breaking down the bulk metal oxide materials into smaller, nanoscale particles. This method contrasts with the bottom-up synthesis, that creates metal oxide nanosheets by assembling smaller molecules or atoms into larger structures. Mechanical exfoliation is one of the most used top-down techniques for producing MO-NSs. This method involves physically exfoliating the bulk metal oxide materials using techniques, such as sonication, grinding, or ball milling [33]. These techniques use mechanical forces to break down the bulk material into smaller, thinner sheets. The resulting metal oxide nanosheets are typically of high quality and have a high degree of crystallinity. Singh et al. [34] proposed a new top-down process to prepare 2D- MnO_2_ nanosheets in stirred media mills. They applied analytical grade manganese dioxide for nano milling experiments, and to increase the stability of the MnO_2_ nanosheet, they employed polyacrylic acid sodium salt. More details about the experiment can be found in Patel et al [35]. 

**Figure 2:**
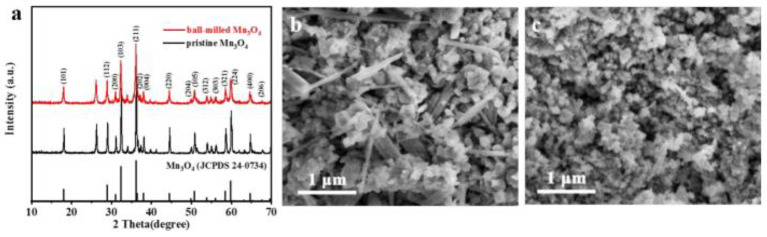
Illustration of the Mn_3_O_4_ characterization process. (**a**) XRD patterns, (**b**) SEM image of the precursor Mn_3_O_4_, and (**c**) of the ball-milled Mn_3_O_4_, respectively. Reprinted with permission from [36]. Copyright 2018 American Chemical Society.

Wang et al. applied ball milling technology to fabricate Mn_3_O_4_ nanoparticles in large quantities for use as a cathode in Zn ion batteries [36]. To form a paste, ethanol was added with Mn_3_O_4_ and by using zirconia milling media the paste was ball-milled for 2 h at a rotational speed of 2000 rpm. Then, the composition was dried to separate C_2_H_5_OH via evaporation at 80 °C following several characterization approaches to obtain Mn_3_O_4_ powder. The pristine and ball-milled Mn_3_O_4_ are shown in Figure 2. 

**Figure 3:**
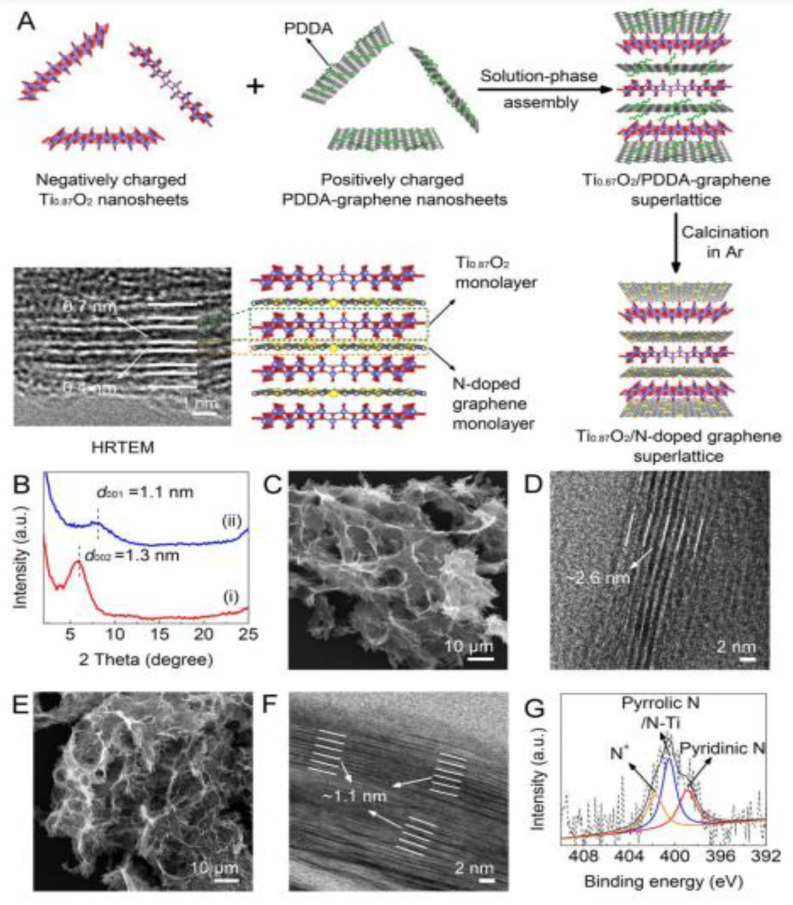
(**A**) Illustration of the synthesis approaches of the Ti0_0.87_O_2_/N-doped graphene superlattice. (**B**) XRD patterns of (i) Ti_0.87_O_2_/PDDA-graphene superlattices and (ii) Ti_0.87_O_2_/N-doped graphene superlattices. (**C**) SEM and (**D**) HRTEM images of Ti0_0.87_O_2_/PDDA-graphene superlattices. (**E**) SEM and (**F**) HRTEM images of Ti_0.87_O_2_/N-doped graphene superlattices. (**G**) High-resolution spectrum of N 1s in Ti_0.87_O_2_/N-doped graphene superlattices. Reprinted with permission from [42]. Copyright 2018 American Chemical Society.

Template-assisted MO-NS preparation is another top-down approach to preparing nanosheets, nanowires, and nanocomposites. Template-assisted MO-NSs fabrication processes are receiving much attention recently and have distinguished themselves as superior to template-free technologies for the fabrication of nanosheets, as they possess improved cycling stability and control over the mechanism, composition, and shape of the finally produced nanosheet. Templates can be classified into two different types, such as soft templates and hard templates [37]. Soft templates, such as NH_4_^+^, amphophilic surfactants, ionic liquids, etc., do not have fixed stiff structures and this results in problems with the morphological product. Moreover, hard templates (colloidal silica spheres, mesoporous materials, microporous zeolites, anodic aluminum oxide membranes, etc.) exhibit good rigidity and stable structures that make these templates promising for utilization [38,39]. Barrer and Denny introduced the templates and templating influences through an experimental study in 196,1 where they prepared zeolites by using tetramethylammonium hydroxide (TMAOH) as the first organic cation [40]. Template-assisted synthesis procedures can be applied for preparing several metal oxide nanosheets for use as energy storage devices. This approach is also applicable to preparing 2D-TiO_2_ which is widely used in Li-ion batteries. Xue et al. experimented with a surfactant-supported exfoliation approach to synthesize 2D-TiO_2_ nanosheets [41]. TiO_2_ nanoparticles were mixed with tetrabutylammonium hydroxide (TBAOH) and reacted at 130 °C for 24 h to formulate a TiO_2_ nanosheet as shown in Figure 3A. The TBAOH not only acted as an alkaline solution but also as an agent to prevent the accumulation of nanosheets (Figure 3B) and the thickness of TiO_2_ was found as 0.40 nm. 

Xiong et al. synthesized Ti_0.87_O_2_ nanosheets via an exfoliation approach and they used layered titanate crystals as raw material [42]. Layered titanate crystals were also applied using this approach and for the same purpose in previous works [43,44]. A solid-state calcination approach was applied to obtain the layered titanate crystals of K_0.8_Ti_1.73_Li_0.27_O_4_. Following stirring K_0.8_Ti_1.73_Li_0.27_O_4_ in HCL solution for 2 days, the protonic form H_1.07_Ti_1.73_O_4_·H_2_O was achieved. Once the T_0.87_O_2_ nanosheet was obtained, it was mixed dropwise under continuous stirring on a hypothetical model from which a Ti_0.87_O_2_/PDDA-graphene superlattice was obtained and this superlattice was annealed for 2 hours at 600 °C to obtain a Ti_0.87_O_4_/N-doped graphene superlattice. The overall process is depicted in Figure 3 that includes several steps of the synthesis process. 

Solid-state grinding is a mechanical process used to produce metal oxide nanosheets. It involves reducing the size of bulk metal oxide materials through the application of physical force. This force can be generated through various methods such as ball milling or mechanical grinding. Anbao et al. developed a novel approach to solid-state grinding reaction at room temperature where they prepared four MnO_2_ nanosheets using Mn(OAc)_2_·4H_2_O and (NH_4_)_2_C_2_O_4_·H_2_O as raw materials and they calcinated MnC_2_O_4_ at four different temperature ranging from 300–600 °C to obtain nano MnO_2_ [45]. Their experimental result revealed that calcination at 400 °C yielded the best result compared to other temperatures.

**Figure 4:**
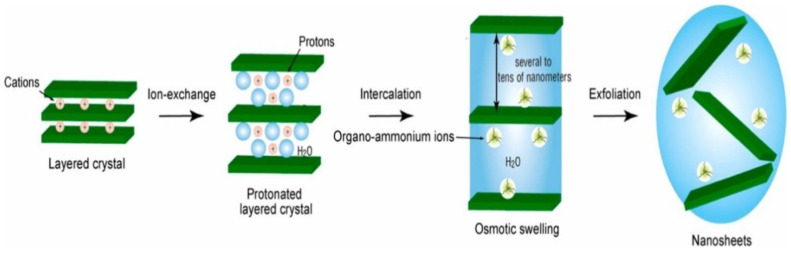
Schematic illustration of the common soft chemical exfoliation approach for synthesizing metal oxide nanosheets. Reprinted with permission from [49]. Copyright 2018 Elsevier.

Another popular top-down method is chemical exfoliation. This method involves the use of a solvent or a chemical to dissolve the bulk metal oxide material and then separate the resulting solution into layers. This method is typically used for metal oxide materials that are not easily exfoliated mechanically. Soft chemical exfoliation is a well-developed process for synthesizing several metal oxide nanosheets such as KCa_2_Nb_3_O_10_, K_0.45_MnO_2_, Cs_0.7_Ti_1.825_O_4_, H_1.07_Ti_1.73_O_4_·H_2_O [46,47,48]. The conventional approach of this process includes four several steps such as synthesis of precursor layered crystals, protonation of the layered crystal through an acid exchange, osmotic swelling, and solution-phase exfoliation which are depicted in Figure 4.

Top-down methods have some advantages over bottom-up approaches. For example, by applying top-down approaches, a huge amount of MO-NSs can be produced, and the resulting nanosheets are typically of high quality and have a high degree of crystallinity. Additionally, top-down methods are generally less time-consuming and less expensive than bottom-up methods. However, top-down methods also have some limitations. For example, the size and thickness of the MO-NSs produced by top-down methods are typically not as well-controlled as those produced by bottom-up methods. Additionally, top-down methods can be more difficult to scale up to industrial-level production. The TBAOH-based nanosheets and their applications in energy storage devices are listed in Table 1.

### 2.2. Bottom-Up Synthesis

**Figure 5:**
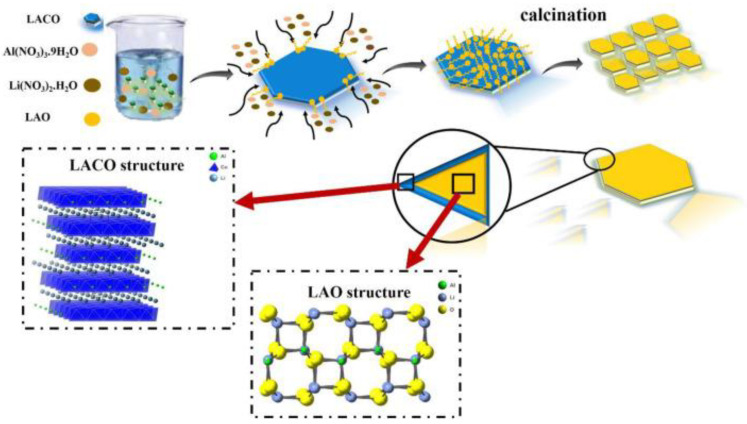
Schematic showing the surface coating process of LAO on LACO. Reprinted with permission from [64]. Copyright 2021 Elsevier.

The bottom-up technologies for synthesizing MO nanosheets (MO-NSs) involve the use of small precursor molecules or atoms to build up the metal oxide structure from the bottom up. The major convenience of this method is that it permits precise control over the composition, morphology, and size of the nanosheets. One of the most common bottom-up methods for the preparation of MO-NSs is the sol-gel method. This method involves the use of metal alkoxides as precursors, which are dissolved in a solvent and then hydrolyzed to form metal oxide nanosheets. The sol-gel method permits scrutinizing control over the structure of the MO-NSs by adjusting the content ratio of the precursors. The sol-gel method is relatively facile and versatile, and it can be employed to produce a massive range of MO-NSs, including oxide nanosheets of transition metals, rare earth metals, and lanthanides. However, the sol-gel approach is usually limited to the production of small quantities of nanosheets. Paydar et al. synthesized LiAl_0.5_Co_0.5_O_2_ by using the sol-gel method where they applied aluminum nitrate (Al(NO_3_)·9H_2_O), Co(NO_2_)·6H_2_O, citric acid, and LiNO_3_ as raw materials with a high purity [64]. Then, by mixing the reacting agent, heating, and drying, the final product (LACO) was found which can be clearly observed in Figure 5.

Another bottom-up method to synthesize the MO-NSs is the chemical vapor deposition (CVD) method. This approach involves the use of metal oxide precursors that are heated to high temperatures in the presence of a gas, causing the metal atoms to react with oxygen to form a thin film of metal oxide [65]. The CVD method also allows for scrutinizing control over the layer thickness, and the structure of the metal oxide nanosheets by adjusting the precursors, and the reaction conditions. However, CVD is a relatively complex and time-consuming method, and it is generally limited to the production of small quantities of nanosheets. Saenz et al. synthesized MoO_x_ nanosheets using the CVD approach where MoO_3_ reacted with sulfur powder precursors, and nitrogen gas was applied as a transport gas [66]. Their prepared material showed high conductivity of nearly 6680.3 S cm^−1^ and an improved bolometric coefficient of 0.152 mS K^−1^. Li et al. synthesized ultrathin MoO_2_ nanosheets on a SiO_2_/Si substrate by using the CVD approach where they employed Mo_2_O_3_ and sublimated S as precursors [67]. Their prepared MnO_2_ nanosheet showed improved conductivity of 3600 S cm^−1^, and thermal stability retained above 200 °C. 

Another bottom-up process for the fabrication of MO-NSs is the electrochemical deposition method. This method involves the use of an electrolyte solution, and a conductive substrate to deposit metal oxide onto the substrate [68]. The electrochemical method can be classified into two categories, namely anodic, and cathodic methods. In the anodic method, metal ions are oxidized to form a metal oxide on the substrate, whereas, in the cathodic method, metal ions are reduced to form metal on the substrate, which subsequently oxidizes to form metal oxide [69]. Kadam et al. reported an electrochemical synthesis approach to prepare a MnO_2_ nanosheet and in their experiment, they applied MnCl_2_·4H_2_O 0.2 m with 99.9% purity as a precursor and stainless steel of 304 grade as the conducting substrate. They annealed the electrodeposited manganese hydroxide at 350 °C for 3 h, which finally resulted in deposited reddish brown colored manganese oxide films [70]. 

Thermoregulated phase transition is an emerging bottom-up synthesis process in which a material changes its state (such as from solid to liquid or gas) as a result of temperature changes. The transition occurs when the temperature of the material reaches a specific threshold, known as the transition temperature, causing the material to rearrange its molecular structure, and exhibit different physical and chemical properties. This process is governed by the intermolecular forces, and thermodynamic properties of the material, and can be influenced by external factors, such as pressure and composition. Zhang et al. proposed a novel process to synthesize MO nanosheets based on the thermoregulated phase transition of the micelles [71]. For forming a solution with string, they mixed deionized water with metal salts, and to prevent hydrolysis of metal salts, they added several acids with it. Then, they dissolved 1.875 Pluronic P123 in the solution to be heated from 10 to 50 °C for 2 h, following the addition of ammonium hydroxide solution and shaken for 1 min. Finally, after several processes, such as centrifugation, washing, and annealing, the MO nanosheets were obtained. Their product showed high retention capacity and high reversible capacity. The electrochemical method allows for precise control over the composition and morphology of the metal oxide nanosheets by adjusting the precursors and the reaction conditions. However, the electrochemical method is generally limited to the production of small quantities of nanosheets.

Zhou et al. proposed a novel ultra-facile route to synthesize several metal oxide nanosheets, such as ZnO, Co_3_O_4_, Fe_2_O_3_, WO_3_, TiO_2_, and so on with a large surface area [72]. To synthesize the ZnO nanosheet in such a typical approach, firstly, the mixture of some precursors, such as Zn(NO_3_)_2_·6H_2_O, urea, and glucose were pre-calcined for 6 h at 140 °C. Then, the pre-calcined mixture was further calcined for 10 h at 500 °C, which finally resulted in ZnO nanosheets. They also noted that the metal oxide nanosheets synthesized in this way can be an attractive potential in energy storage applications.

Overall, the bottom-up approach to fabricating MO-NSs is a powerful method to prepare high-quality MO-NSs with precise control over their properties. The potential applications for metal oxide nanosheets are vast and varied, and the development of new bottom-up synthesis methods will continue to expand the possibilities for their use in a wide range of fields. Different methodologies for nanosheet synthesis with their advantages and limitations are listed in Table 2 for comparison.

## 3. Applications of MO Nanosheets in Batteries

### 3.1. MO Sheets Used in Li-S Batteries

Lithium sulfide batteries are increasing in popularity due to their massive convenient applications as electrochemical energy storage devices. During the transformation of sulfur (S) into sulfur ion (S^−2^), a high capacity (1675 mAh g^−1^) can be obtained, which is almost ten times that of commercial electrodes that are applied in lithium-ion batteries [73]. Sulfur possesses a much lower redox potential that allows these materials to enhance the operating voltage and energy density combined with alkali metals [74]. A high energy density, such as 2600 Wh kg^−1^, can easily be achieved by using a lithium-sulfur battery [75]. There are also some conveniences and limitations of sulfurs that can influence a Li-S battery. Among the conveniences, availability and biocompatibility are to be noted, while the inactivity of big particles caused by lower conductivity is considered the major barrier of sulfur-assisted batteries [76]. Another major limitation is the massive volume change during charging and discharging, which reduces the performance of a battery [77]. The cycle lifetime, which originated from the lithium-polysulfide (LIPS) shuttling process, is a major barrier of Li-S batteries. Researchers have taken a lot of approaches and have developed several systems to control this shuttling effect, but they have not found potential solutions for this. To approach an effective solution for this limitation, Patil et al. [78] synthesized a 2D lepidocrocite TiO_2_ nanosheet to apply in sulfur cathodes, which could minimize the polysulfide dissolution significantly. For this, the surface area was also improved, and the Li-S cell possessed 1023.5 mAh g^−1^ at 50 mA g^−1^ for just 80 wt% sulfur content. The capacity was also improved and after 300 cycles, an 82.3% capacity was retained.

### 3.2. MO Nanosheets for Zn-ion Batteries

Metal oxide nanosheets have emerged as an promising material to utilize in zinc-ion batteries, due to possessing some attractive features, such as a large surface area, high energy density and extradentary electrochemical properties, and low cost [79,80,81]. Zinc-ion batteries are being considered as a potential alternative to traditional lithium-ion batteries, due to the abundance and low cost of zinc, as well as the lack of safety concerns associated with lithium [82]. The large surface area of the nanosheets allows for the more efficient storage of zinc ions, leading to a higher capacity and better performance of the battery. Additionally, the high surface area also increases the rate of charge and discharge, making the battery more efficient. Another advantage of metal oxide nanosheets is their excellent electrochemical properties. Metal oxide nanosheets have been shown to have good conductivity, stability, and reversibility, which are all important factors for the performance of zinc-ion batteries. Additionally, the use of metal oxide nanosheets can improve the overall safety of the battery, as they have been shown to have a high thermal stability and good mechanical properties.

**Figure 6:**
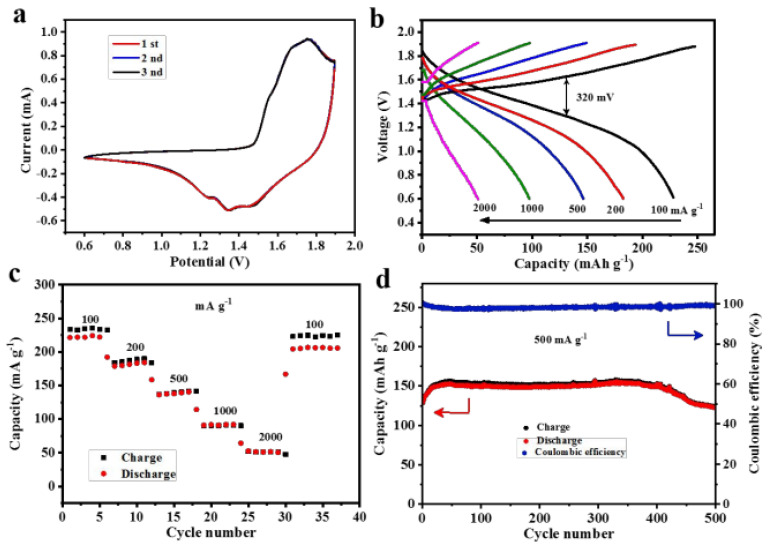
Electrochemical performance of the Zn/ε-MnO_2_ coin cells. (**a**) Cyclic voltametric (CV) curves of the Zn/ε-MnO_2_ cell at a scan rate of 0.1 mV s^−1^. (**b**) The discharge/charge voltage profiles at various current densities between 0.6 and 1.9 V. (**c**) Rate capability. (**d**) Long-term cyclic performance and the corresponding Coulombic efficiency at 500 mA g^−1^. Reprinted with permission from [35]. Copyright 2018 American Chemical Society.

Different types of metal oxide nanosheets have been proposed for use in zinc-ion batteries. These include zinc oxide, titanium dioxide, and iron oxide nanosheets [83,84]. Each of these materials has its unique properties and advantages, and researchers are currently investigating which of these is the most promising for use in zinc-ion batteries. Zn/MnO_2_ batteries are also considered emerging in recent years due to their low cost, environmental benignity, scalable preparation, and safety. Wang et al. carried out a novel approach to prepare MnO_2_ from Mn_3_O_4_ based on the ball milling technology to utilize the product in Zn-ion batteries. The transformed akhtenskite MnO_2_ had a high retention capacity above 92% (after 500 cycles at 500 mA g^−1^) and a high reversible capacity of 221 mAh g^−1^ (at 100 mA g^−1^). In the meantime, the cathode showed a high specific energy of nearly 288 Wh kg^−1^, which is much higher compared to the available Pb-acid batteries [35]. They also found a small self-discharge limitation of their battery, but the source of this issue was unclear to them. However, they reported that concerning the long-time energy storage demand, their battery could be applied as a promising device. Figure 6 depicts the electrochemical performance of the Zn/ε-MnO_2_ cell, where Figure 6a shows cyclic-voltammetry (CV) curves between 0.6 to 1.9 V, Figure 6b shows a pair of redox peaks (at 1.7 and 1.32 V), and Figure 6c depicts the rate capacity of the transformed Zn/ε-MnO_2_ cathode, while the current density increased steadily from 100 to 2000 mA g^−1^. 

Vanadium-based oxides are gaining much attention for application in Zn-ion batteries, as they are environmentally friendly, easily available, and have a high specific capacity [85]. Liang et al. prepared a Ni-doped M_x_O_y_ nanosheet for application in Zn ion batteries, and to form oxygen vacancies, they applied H_2_ annealing processes [86]. Their prepared device showed excellent performance with a capacity of 0.68 mAh cm^−2^, a current density of 2 mA cm^−2^, and capacity retention of over 6000 cycles. It also showed a high power and energy density of 3.34 mW cm^−2^ and 1.13 mW cm^−2^, respectively. 

Co_3_O_4_ nanosheets are emerging materials for Zn-ion batteries. Wang et al. prepared Co_3_O_4_@Ni to use as a positive electrode in Zn//Co_3_O_4_ and they applied KOH as an electrolyte [87]. Their device provided a high energy density of 241 Wh kg^−1^ and revealed a high cycling stability. Zinc-manganese oxide nanosheets are very popular as electrodes for Zn-ion batteries. Zhang et al. reported ZnMn_2_O_4_ as an anode material to investigate its performance in Zn-ion batteries, where they applied Zn(CF_3_SO_3_)_2_ as an electrolyte [88]. Their system revealed excellent performance with a reversible capacity of 150 mAh g^−1^ and a retention capacity of 94% after 500 cycles.

### 3.3. MO Nanosheets for Zn-air Batteries

Zinc-air batteries can provide a potential solution where it is required for large-scale energy storage because Zn-air batteries have some attractive features, such as stability, durability, low expense, safety, high power, and energy density. Li et al. reported a nickel-doped CoO nanosheet as electrode material in Zn-air batteries for the first time and their battery showed great performance with a high power density of 377 mW cm^−2^, and it worked for more than 400 h with a high stability [89]. They also compared their battery with a Pt/C catalyst device and revealed that Ni-doped CoO nanosheets outperformed, based on the charge/discharge voltage. Tian et al. prepared oxygen defective amorphous crystalline CoO (ODAC-CoO) nanosheets from Co(OH)_2_ by using a vacuum-calcination approach and they found that their sample possessed an improved oxygen reduction reaction and oxygen evolution reaction, and it also showed a high stability [90].

To improve the retention capability and performance of Zn-air batteries, a bifunctional O_2_ catalyst plays a pivotal role. MnO_2_ is considered the most efficient material to apply as a catalyst in Zn-air batteries to improve the ORR. Zhong et al. employed a facile solution-based approach to modify MnO_2_-NS with nickel, cobalt, or iron [91]. They reported that by taking such an approach, they were able to enhance the ORR and OER for the Co-MnO_2_ sample, and it exhibited an improved power density of 167 mW cm^−2^.

### 3.4. MO Nanosheets for Li-Ion Batteries

**Figure 7:**
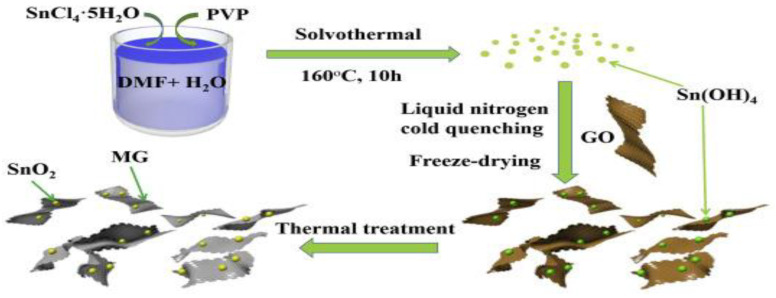
The synthesis process of SnO_2_/MG nanosheets. Reprinted with permission from [96]. Copyright 2019 Elsevier.

Lithium-ion batteries (LIBs) are competitive options for EESs in the clean energy market and a lot of research has already been carried out due to their applicability as energy storage devices [92,93,94]. To ameliorate the Li-ion batteries, Zhang et al. prepared a V_2_O_3_@NC nanosheet to utilize as a free-standing cathode in a lithium-ion battery, and through TEM images, it was investigated that the V_2_O_5_ nanoparticles were layered homogeneously in the carbon region [95]. The charge and discharge specific capacities were found as 508 and 984 mAh g^−1^, respectively, and the reversible capacity after 400 cycles was found as 892 mAh g^−1^ at 0.1 A g^−1^. Li et al. prepared a GN/SnO_2_ nanosheet via a facile cold-quenching approach and reported that, by having the distinctive wrinkled surface property, the maintenance of the monodispersed state was facilitated. Their hybrid nanosheet material exhibited a high reversible capacity of 1147 mAh g^−1^ at 100 mA g^−1^ [96]. The synthesis process of SnO_2_/MG nanosheets is illustrated in Figure 7.

Li et al. prepared a new 2D LiNi_1/3_Co_1/3_Mn_1/3_O_2_ nanosheet by using sol-gel technology [97]. They found that their prepared material showed high performance as a cathode and it showed a high discharge capacity of 137.7 mAh g^−1^ at 20C. Mei et al. [98] synthesized 2D Bi_2_O_3_ nanosheets for the first time by applying a solution-based self-assembly process and their nanosheet material supplied a high capacitance above 200 mAh g^−1^ and discharge capacity of 647.6 mAh g^−1^, when utilized as an anode in a Li-ion battery. Li et al. used an electrodeposition technique to synthesize a two-dimensional CoO nanosheet and found that their nanosheet possessed extraordinary performance with a high retention capacity of 1000 mAh g^−1^ at 1 A g^−1^ over 100 cycles [99].

### 3.5. NO Nanosheets for Na-ion Batteries

**Figure 8:**
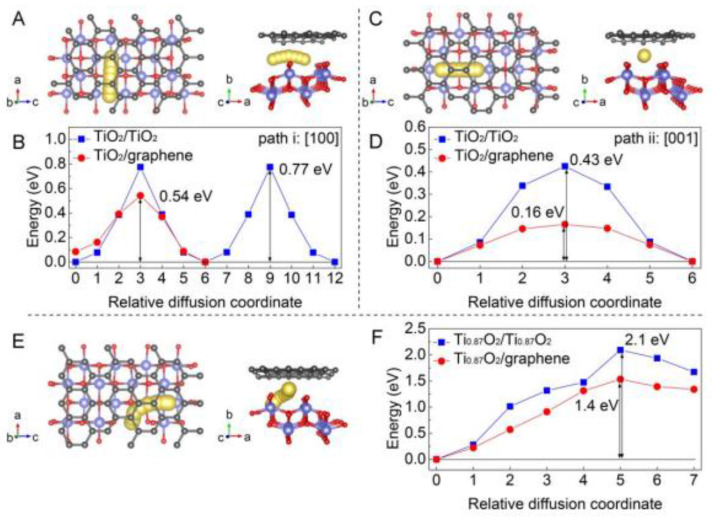
(**A**) Diagram of the sodium diffusion path towards the [100] directions between the TiO_2_/graphene bilayers. (**B**) The energy of sodium diffusion along the [100] direction in TiO_2_ bilayers and TiO_2_/graphene bilayers. (**C**) Illustration of the Na diffusion path along the [001] directions between the TiO_2_/graphene bilayers. (**D**) The energy of Na diffusion along the [001] direction in TiO_2_ bilayers and TiO_2_/graphene bilayers. (**E**) Illustration of a possible Na diffusion path in Ti_0.87_O_2_/graphene bilayers. (**F**) The energy of Na diffusion in Ti_0.87_O_2_ bilayers and Ti_0.87_O_2_/graphene bilayers. Reprinted with permission from [41]. Copyright 2018 American Chemical Society.

Xiong et al. reported a Ti-deficient 2D Ti_0.87_O_2_ nanosheet superlattice for application in Na-ion batteries [41]. They also reported that Ti_0.87_O_2_ with a N-doped graphene monolayer possessed attractive performance, that could be applied in Na-ion battery with a high capacity of nearly 490 mAh g^−1^ at 0.1 A g^−1^. The simulation results, which are shown in Figure 8, reveal conspicuously a lower diffusion barrier of energy for diffusing Na^+^ ions in Ti_0.87_O_2_/graphene, demonstrating its superiority for Na ion batteries. 

Rubio et al. synthesized a hybrid MO nanosheet utilizing iron oxide and iron sulfide, to apply as an anode in Na-ion batteries [100]. There are also some previous studies where researchers worked with these two materials as an anode, but the materials had some disappointing properties when they were applied for battery applications [101,102]. At the time of charging and discharging, these materials showed an excessive fluctuation in volume, and another limitation of these materials was electron fragility, which limited their applications in electrochemical storage devices [103,104,105]. 

Chen et al. prepared a metal oxide anode for application in a sodium ion battery and MO K_0.8_Ti_1.73_Li_0.27_O_4_ (KTLO) was fabricated via a simple flux process that is based on ball-milling [57]. The KTLO, as a Na-ion battery, showed a high specific capacity of 119.6 mAh g^−1^ at 20 mA g^−1^ with a high retention capacity for more than 250 cycles. The carbon-coated KTLO enhances the structure stability and electronic conductivity which makes this material an attractive anode material for sodium-ion batteries. 

Li et al. prepared a GN/SnO_2_ nanosheet via a facile cold-quenching approach and reported that by having the distinctive wrinkled surface property, the maintenance of the monodispersed state was facilitated [96]. Their hybrid nanosheet material exhibited a high reversible capacity of 314 mAh g^−1^ at 100 mA g^−1^.

Sun et al. prepared a two-dimensional FeO_x_ nanosheet and applied it as an anode in Na-ion batteries [106]. They reported that their synthesized MO nanosheet showed an attractive output with a mean specific capacity of 408 mAh g^−1^ at a current density of 0.21 A g^−1^. Further application of their electrode revealed that after completing 100 cycles, the specific capacity was reduced to 263.4 mAh g^−1^. They also showed that amorphous FeO_x_ nanosheets possessed a faster electron movement compared to crystalized Fe_3_O_4_ nanosheets, that showed a better capacity for FeO_x_ than for Fe_3_O_4_. 

### 3.6. Battery-Supercapacitor Hybrid (BSH) Device

**Figure 9:**
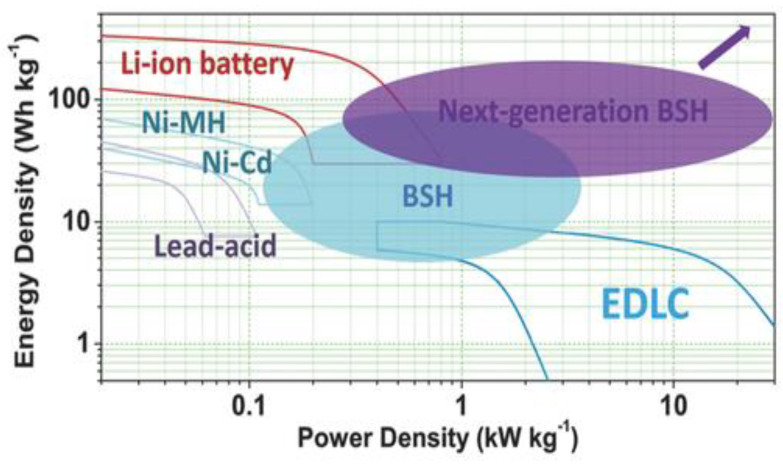
Ragone plots of various rechargeable batteries and EDLC, and the comparison with BSHs. Reprinted with permission from [110]. Copyright 2017 Willey online library.

For achieving a higher performance and cost-effective energy storage systems, researchers have developed several hybrid electrochemical energy storage technologies, such as battery-supercapacitor hybrid systems (BSH) [107]. A BSH system combines the advantages of batteries and supercapacitors by building battery-based and supercapacitor-based electrodes. Figure 9 demonstrates that Ni-MH and Ni-Cd batteries, which have been utilized in secondary batteries for over a century due to their greater specific capacities, are still commonly used today, and have an energy density of 30–70 Wh kg^−1^ [108]. By using inorganic carbon hybrid electrodes, recently developed Ni-Fe alkaline batteries can also charge extremely quickly and can deliver specific energy densities greater than 100 Wh kg^−1^ [109].

**Figure 10:**
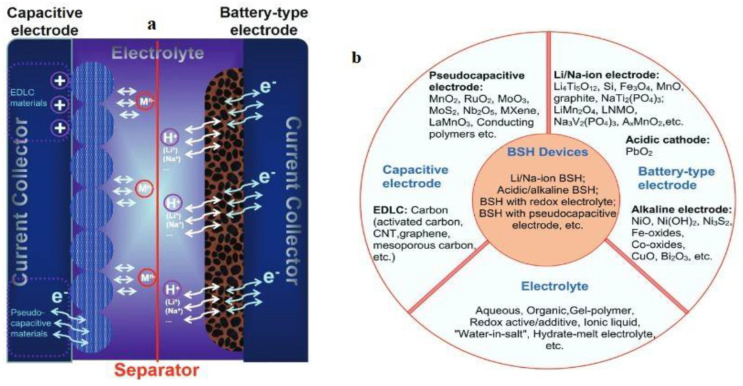
(**a**) A typical hybrid energy storage device with a working process. (**b**) Different types of hybrid devices and their electrolyte and electrode materials. Reprinted with permission from [110]. Copyright 2017 Willey online library.

A typical BSH energy storage device is depicted in Figure 10a, where anions and cations migrate to the two electrodes during charging and discharging, where the battery electrode undergoes the redox reaction, and the Sc electrode accumulates ions. There are lots of conveniences to modeling different types of BSH devices, such as diverse types of electrodes, device configurations, and electrolytes. The potential candidate for electrodes and electrolytes in BSH devices are captured in Figure 10b. 

**Figure 11:**
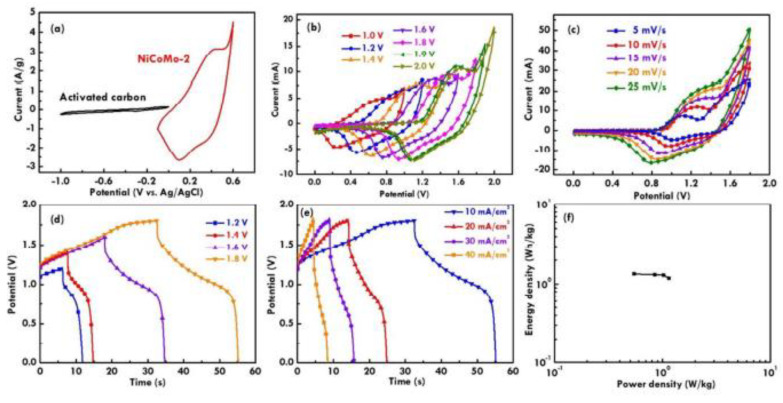
The current-potential curves for (**a**) the anodes and cathodes, (**b**) the current-potential curves measured using several potential windows, (**c**) different scan rates, (**d**) the GC/D plots measured using several potential windows, (**e**) different current densities, and (**f**) the Ragone plot of the BSH. Reprinted with permission from [111]. Copyright 2018 American Chemical Society.

Huang et al. [111] developed such BSH devices that included activated carbon as the anode and the Ni_x_Co_y_Mo_z_O as a cathode. They measured the optimized potential window for the anode and cathode. Figure 11a shows the current-potential curves measured for both electrodes, and Figure 11b illustrates the current-potential curves of the cathode and anode using several potential windows, and it revealed that a window with 1.8 V showed the best scan rates. Figure 11d,e illustrate the GC/D plot which also suggests a window of 1.8 V for application in hybrid devices. They found power and energy densities of 3.5 W kg^−1^ and 22.02 Wh kg^−1^, respectively, and at a current density of 10 mA cm^−2^, they found a C_F_ value of 126 mF cm^−2^. Figure 11f illustrates the Ragone plot of the BSH.

Researchers have developed several Li-ion-based, Na-ion-based, alkaline, and acidic BSH systems which have been increasing in popularity in recent years. A brief comparison is carried out in Table 3 for a proper understanding of different BSH devices.

## 4. Applications of Metal Oxide Nanosheets for Fuel Cells

### 4.1. Solid Oxide Fuel Cell

**Figure 12:**
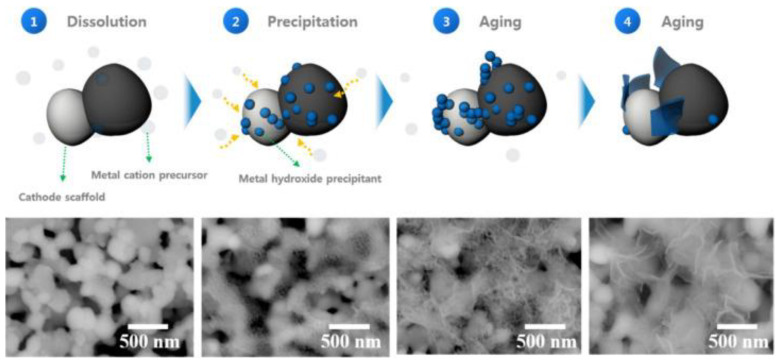
Schematic of the step-wise synthesis process of the catalyst and corresponding SEM images. Reprinted with permission from Ref. [134]. Copyright 2021 Elsevier.

Solid oxide fuel cells (SOFCs) are the type of cells that can generate energy by utilizing hydrogen fuel, and in recent years with the increasing amount of hydrogen fuel generation, the application of SOFCs is increasing steadily [125,126]. The major limitation of these fuel cells for application is their poor reliability at high temperatures due to the thermal corrosion in their components [127,128]. In this regard, lowering the cell operating temperature cannot be a potential solution as at a lower temperature the charge conductivity of the cathode decreases. To find a potential solution, researchers have developed cobalt (Co) containing perovskite, which possesses high charge conductivity [129,130,131,132]. Kim et al. prepared a SOFC device using a La_0.6_Sr_0.4_CoO_3 − δ_ nanosheet which facilitated the conduction of charge in the cathode material [133]. They prepared the MO nanosheet in several steps (Figure 12) and found that the cell power density enhanced to 1.2 W cm^−2^ at 600 °C. 

### 4.2. Direct Methanol Fuel Cell

**Figure 13:**
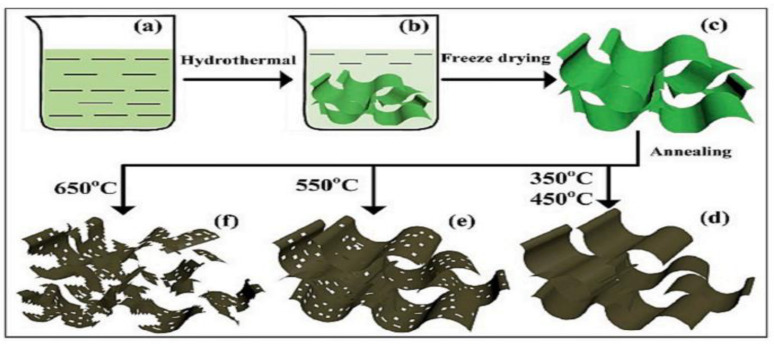
Synthesis flow diagram of NiO. Reprinted with permission from [134]. Copyright 2021 Elsevier.

Jia et al. synthesized an ultra-thin 2D NiO nanosheet to use as an electrocatalyst in direct methanol fuel cell devices [134]. They prepared this NiO nanosheet via a hydrothermal approach with four several steps as shown in Figure 13. They tested the NiO precursor at four different annealing temperatures ranging from 350 to 650 °C and gave those names as 350-NiO, 450-NiO, 550-NiO, and 650-NiO respectively. They found that with increasing annealing temperature, the surface area of this nanosheet decreases, which causes a deterioration in the performance of the catalyst. In this regard, NiO at 350 °C annealing temperature showed the best catalytic performance compared to NiO at other temperatures.

TiO_2_ nanosheets with Pt-derived catalysts are increasing in popularity, for utilization in direct methanol fuel cells. Saida et al. experimented with TiO_2_ applicability in a methanol fuel cell where they synthesized TiO_2_ nanosheet from layered H_2_Ti_4_O_9_ and then mixed the product with PtRu/C catalyst, which was prepared by the impregnation method in disparate compositions. The modified composite catalyst enhanced the electrooxidation of CO_2_ and methanol, which caused a rise in the electrolyte and PtRu catalyst. The TiO_2_ nanosheet-modified PtRu/C showed higher ECSA compared to unmodified PtRu/C [54]. 

### 4.3. PEM Fuel Cells

The proton exchange membrane (PEM) fuel cell is the most widely applied fuel cell and in recent years, researchers have focused on it due to its higher capacity compared to other electrochemical energy storage devices. The working mechanism of the PEM fuel cells includes the transformation of the proton from the anode side to the cathode side and the electricity is produced during the half-reaction. There are some attractive conveniences, such as no vibration, no mechanical parts, and no CO_2_ emissions during operation with H_2_ as fuel, and this system is also suitable for various temperature ranges. In recent years, researchers have felt the requirement for proper electrocatalyst materials that can replace Pt and Pb-based catalysts to obtain a clean energy storage system [135,136,137]. In this regard, they found the MO nanosheet to be a suitable material that can be applied as an electrocatalyst due to its large active sites, and attractive mechanical properties that can enhance the capability of a speedy charge transfer between two electrodes [138]. Tiido et al. reported Pt/TiO_2_ -graphene as an attractive and potential cathode catalyst for application in PEM fuel cells [139]. In their experiment, they prepared TiO_2_ functionalized graphene nanosheets to employ as support for Pt nanoparticles. Their microscopic and electrochemical investigation revealed that the electrode exhibited a high electrocatalytic performance. 

**Figure 14:**
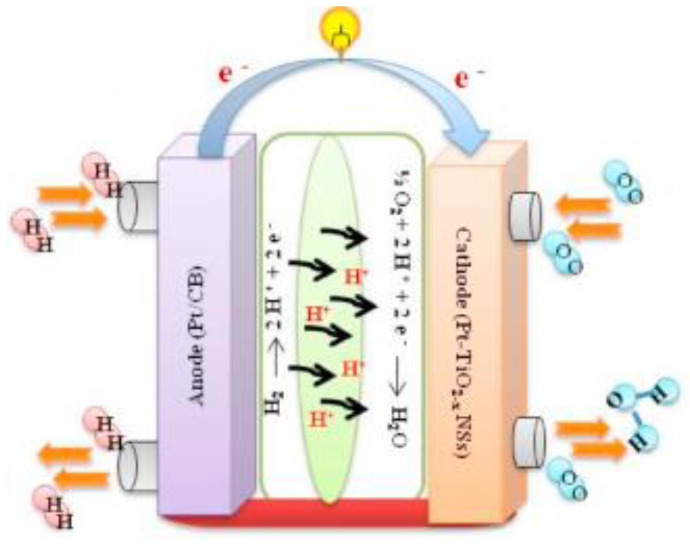
Schematic representation of a PEM fuel cell [140]. Copyright permission 2020, Elsevier.

**Figure 15:**
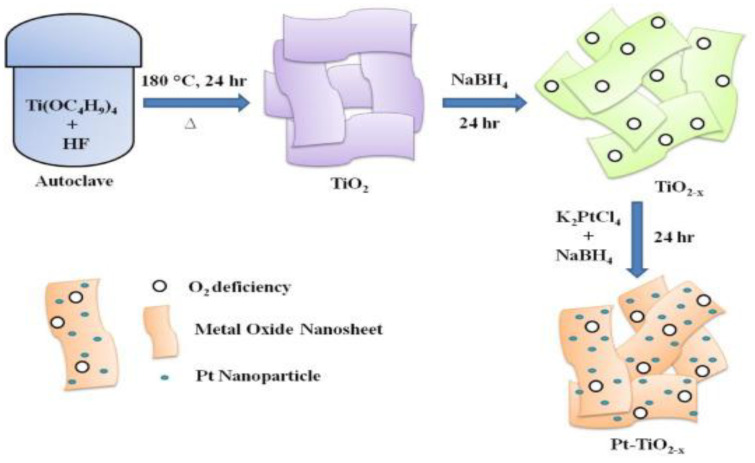
Schematic illustration of the preparation of Pt–TiO_2 − x_ NS. Reprinted with permission from [140]. Copyright 2020 Elsevier.

Naik et al. developed an oxygen-deficient TiO_2_ nanosheet to apply as a supporting material catalyst in PEM fuel cells, as shown in Figure 14. In addition, they found that Pt-TiO_2 − x_NS, as illustrated in Figure 15, showed extraordinary performance to improve the stability and activity of oxygen reduction reaction [140]. They reported that TiO_2 − x_NS to be a promising support that could be used in future PEM fuel cells, as its performance was experimentally proved. They carried out a square-wave potential cycle test at 80 °C with their catalyst support and found that it possessed durability over 10,000 cycles and excellent stability for more than 100 h. Pt-NiO_2 − x_NSs assisted fuel cells showed a higher power density of 958 mW cm^−2^, as compared with Pt-C catalysts.

In addition to the above-mentioned metal oxide nanosheets, other metal oxides, such as cobalt oxide (Co_3_O_4_), manganese oxide (MnO_2_), and vanadium oxide (V_2_O_5_), have also been studied as catalysts for PEM fuel cells. These metal oxide nanosheets have been found to have a high surface area and good electronic conductivity, which can improve the performance and durability of PEM fuel cells. Lejing et al. reported that Co_3_O_4_ applied in a PEM fuel cell can enhance the oxygen evolution reaction (OER) [141]. They investigated Co_3-x_Ce_x_O_4_ and Co_3_O_4_ nanosheets to compare their potential in electrodes and found that the first material had less potential to reach a current density of 100 mA cm^−2^ and the addition of Ce resulted in the exposure of more active sites. 

### 4.4. Microbial Fuel Cells

**Figure 16:**
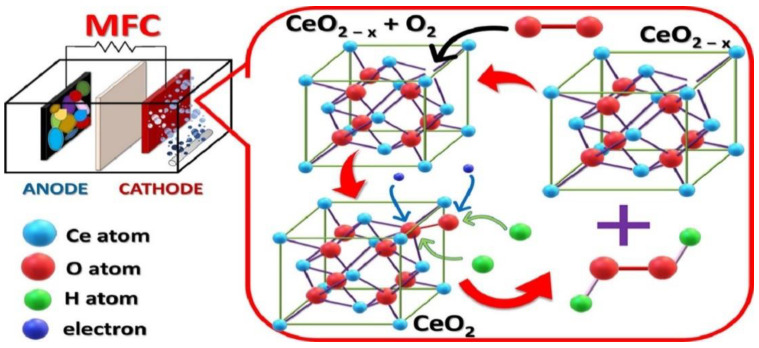
Schematic diagram of a microbial fuel cell. Reprinted with permission from [147]. Copyright 2021 Elsevier.

Over the last decade, the application of microbial fuel cells (MFCs) has risen with proper understanding related to their structure, working procedure, and advancement strategies [142]. In regards to MFC, some researchers have been more conscious of the power that this system consumes, others have been worried about its applications, other researchers identify it as a toxicity conveyer, and it has also been considered as a system through which wastes can be utilized while ignoring environmental pollution [143,144]. Moreover, H_2_O_2_ is considered a pivotal element for various applications that can be generated through MFC when ORR is facilitated by a two-electron mechanism instead of the four-electron [145,146]. Chakraborty et al. synthesized a multilayer CeO_2_ nanosheet to investigate its performance as a cathode material in a dual chamber MFC (Figure 16) [147]. They aimed to minimize the reduction of O_2_ to H_2_O_2_ in the cathode and their result revealed that MFC with CeO_2_ as catalyst showed the highest yield of H_2_O_2_ of nearly 221.4 mg L^−1^ in one day compared to the bare C electrode that yielded nearly 133.4 mg L^−1^ H_2_O_2_ in similar operating conditions.

One of the metal oxide nanosheet that have been studied for use in MFCs is titanium dioxide (TiO_2_) nanosheets. TiO_2_ nanosheets have been found to have a high surface area, which can increase the amount of active material that can be loaded onto the anode. Additionally, TiO_2_ nanosheets have been found to have good electronic conductivity, which can improve the rate performance of MFCs. Research has shown that the use of TiO_2_ nanosheets can significantly improve the power density and durability of MFCs by increasing the microbial adhesion and reducing electron transfer resistance. Yin et al. suggested the application of composite materials in electrodes that can enhance the performance of electrodes via complementary elements [148]. For this purpose, they studied modified conductive polyaniline (PANI) TiO_2_ nanosheets (TiO_2_-NSs), where they deposited disparate amounts of PANI on TiO_2_-NSs during different cycles, such as 5, 10, 15, 20, and 25. Their result revealed that 63.6% of power density can be increased by using TiO_2_-20PANI/CP (813 mW m^−2^) compared to a TiO_2_-NSs/CP anode.

Another type of metal oxide nanosheet that has been investigated for use in MFCs is nickel oxide (NiO) nanosheets. NiO nanosheets have been found to have a high surface area and good electronic conductivity, which can improve the performance of MFCs. Research has shown that the use of NiO nanosheets can significantly improve the power density and durability of MFCs by increasing the microbial adhesion and reducing electron transfer resistance. Furthermore, NiO nanosheets have been found to have a high electrocatalytic activity for the oxygen reduction reaction (ORR), which is an important reaction in MFCs. This increased ORR activity can lead to improved MFC performance. Huang et al. [149] took the first attempt to apply NiO/CNT as a cathode catalyst and reported that 77% NiO/CNT showed maximized power density of 670 mW m^−2^. They also stated that increasing the growth of the content of NiO in CNT, steadily enhanced the performance of MFCs.

Another MO-NS that has been studied for use in MFCs is manganese oxide (MnO_2_) nanosheets. MnO_2_ nanosheets have been found to have a large surface area, good electronic conductivity, and a high electrocatalytic activity for the ORR, which makes them a promising material for use in MFCs. Research has shown that the use of MnO2 nanosheets can significantly improve the power density and durability of MFCs by increasing microbial adhesion, reducing electron transfer resistance, and increasing the ORR activity. Zhang et al. synthesized a mesoporous layered α-MnO_2_ nanosheet of 2 nm to maximize the performance by utilizing the conveniences of porous materials and 2D nanosheets [150]. During its application as an ORR catalyst in MFC, it showed excellent electrocatalytic performance compared to conventional Pt/C as α-MnO_2_ exposed an ultra-large surface area of 339 m^2^ g^−1^. CoO was reported as a potential catalyst for microbial fuel cells by Huang et al [151]. They prepared a catalyst of CoO nanosheets which was supported by N-doped activated carbon and their prepared nanocomposite exhibited low total resistance of 9.26 Ω, 4-electron ORR pathway, and a large surface area which made them preferable for application in MFCs as a cathodic catalyst.

## 5. Application of MO Nanosheets in Supercapacitors

Primary metal oxide nanosheets are ultra-thin two-dimensional sheets made up of metal oxide materials. They have thicknesses in the order of a few nanometers and are considered to be a type of nanomaterial due to their small size and unique properties. They have potential applications in several fields, such as energy, electronics, and catalysis. The properties of metal oxide nanosheets can be tailored by controlling their thickness, surface area, and defects, leading to potential opportunities for new and improved technologies. Primary metal oxide nanosheets are suitable for supercapacitor applications as they possess a large surface area and electrochemical stability. The large surface area of the nanosheets supplies ample space for the storage and transfer of electrical charge, making them highly effective in energy storage. Additionally, the metal oxide materials used in the nanosheets have a high stability, preventing degradation and ensuring long-term performance in energy storage applications. These properties make primary metal oxide nanosheets an attractive option for use as electrodes in supercapacitors, where they can deliver high energy density and a long cycle life.

Rubidium oxide (RuO_2_) nanosheet is considered a potential and attractive element to apply in supercapacitors due to its high electrical conductivity and capacitance. RuO_2_ derived from layered ruthenic acid hydrate (H_0.2_RuO_2.1_.nH_2_O through exfoliation approach and experimental testing revealed that this RuO_2_ nanosheet had a high specific capacitance of 658 F g^−1^ that is ten times higher compared to rutile type RuO_2_ [152,153]. 2D MnO_2_ nanosheets are also attractive materials that can be applied as an electrode in supercapacitors. Research has been carried out on approaches to increase the performance of this type of electrode and has tested the intercalation attitudes of different metal ions, such as Na^+^, Mg^2+^, Li^+^, and K^+^ in layered MnO_2_ [154]. Two-dimensional MnO_2_ nanosheets are also attractive materials that can be applied as an electrode in supercapacitors. Research has been carried approaches to rise the performance of this type of electrodes and tested intercalation attitudes of different metal ions such as Na^+^, Mg^2+^, Li^+^, and K^+^ in layered MnO_2_ [154]. 2D MnO_2_ nanosheet as an electrode of supercapacitor showed an attractive specific capacitance of 171 F g^−1^ at a current density of 3 A g^−1^ [155]. Jin et al. prepared a MnO_2_-Ti_3_C_2_ nanosheet to investigate its performance and compared it with the capacitance and performance of MnO_2_-rGO nanohybrids [156]. The porosity and ion diffusivity of Ti_3_C_2_ was found to be higher than that of the rGO-assisted nanosheet and the specific capacitance and surface area of MnO_2_-Ti_3_C_2_ were also greater compared to the other material.

**Figure 17:**
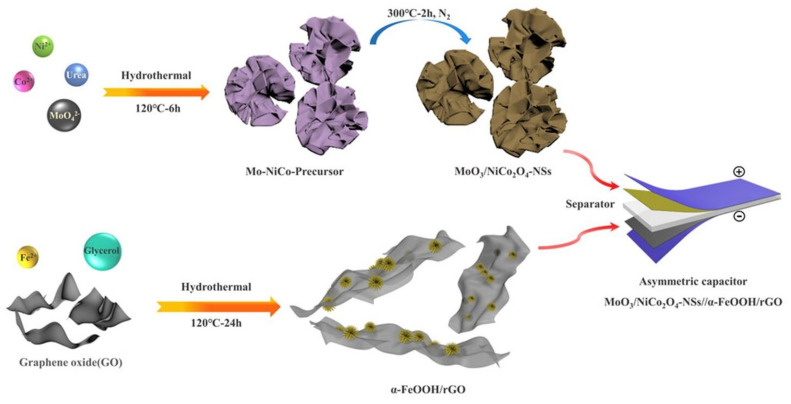
Schematic of MoO_3_/NiCo_2_O_4_-NSs, α-FeOOH/rGO synthesis (hydrothermal) process, and asymmetric supercapacitor. Reprinted with permission from [157]. Copyright 2019 Elsevier.

To enhance the performance and capacitance of supercapacitors, Lin et al. prepared a MoO_3_/NiCo_2_O_4_ nanosheet electrode to apply as cathode material, and α-FeOOH/rGO as anode material and they applied a hydrothermal approach (Figure 17) to synthesize these materials [157]. The cathode showed an attractive specific capacitance of 1042 F g^−1^ at a current density of 1 A g^−1^ and the retention capacity was 110.8% after 2000 cycles. 

Peng et al. proposed that NiMoO_4_ nanosheets exhibited a higher capacitance compared to NiMoO_4_ nanorods based on their application as electrodes for supercapacitors [123]. They prepared an asymmetric supercapacitor with activated carbon as anode and NiMoO_4_ as cathode and found that their device showed an excellent electrochemical property with a high-power density of 850 W kg^−1^ and energy density of 60.9 Wh kg^−1^. Moreover, the device retained 85.7% after 10,000 cycles which showed its stability and durability. 

Researchers have developed another type of supercapacitor that is a flexible fiber-shaped supercapacitor that has become popular due to its portability and wearable electronics. However, these devices cannot provide much operating voltage, and the specific capacitance is not high, which limits their wide application and use. Neng et al. prepared an asymmetric supercapacitor (ASC) where they applied a MnO_2_ nanosheet as a cathode and carbon fiber as an anode [158]. Their ASC showed an extraordinary performance with a high energy density of 27.2 Wh kg^−1^, a specific capacitance of 87.1 F g^−1^, and 95.2% capacitance retention after 3000 cycles. 

Micro supercapacitors (MSCs) are also emerging EES devices with some attractive conveniences, such as high specific capacitance, less expensive, thin atomic layers, etc., and MO nanosheets are an appropriate material as an electrode for MSCs. Wang et al. prepared one such MSC device based on δ-MnO_2_ nanosheet ink [159]. On glass and polyimide film substrates treated with O_2_ plasma, a highly concentrated ink comprising 2D δ-MnO_2_ nanosheets was inkjet printed to create δ-MnO_2_ shapes without the negative “coffee ring” effect. Their device showed extraordinary performance with a power density of 0.018 W cm^−3^ and energy density of 1.8 × 10^−4^ Wh cm^−3^, capacitance retention of 88% over 3600 cycles. Applications of several MO nanosheets in different EES systems are given in Table 4.

## 6. Challenges and Prospects

Metal oxide nanosheets have emerged as potential elements for enhancing electrochemical energy storage systems because of their extraordinary physical and chemical features. Researchers have proved their potential as a catalyst in fuel cells and electrodes in batteries and supercapacitors. However, the major concern is the limitation of large-scale production of MO nanosheets that restricted their production and applications. Following a review of the previously published works, we came to realize that the other limitation is the limited power and energy density of MO-NS-based energy storage technologies. Therefore, further study and research is required to utilize MO nanosheets in energy storage technology. Moreover, more research efforts are required to obtain materials with more reliability, stability, and composition tuneability via eco-environmentally friendly and facile approaches. Several additives and nanocomposites should be derived to enhance the performance of various MO-NSs. 

There are also some prospects in the utilization of metal oxide nanosheets for EESs that should be noted.

1. Researchers are performing a lot of studies and experiments for synthesizing novel MO nanosheet materials with improved physical and electrochemical properties, such as enhanced cyclic stability, high retention capacity, high power and energy density, and speedy charging-discharging rates. Additionally, researchers have also carried out improvements in hybrid MO nanosheets which may provide better performance in the near future compared to the nanosheets prepared up to now;
2. MO nanosheets can be utilized in several electrochemical energy storage devices, conveying the aim that these devices can capture and store renewable energy in a portable device for further use. This concept inspires us to utilize renewable energy sources and particularly, this can negate the application of carbon in supercapacitors and can make the energy system more reliable and environmentally friendly in the near future;
3. MO nanosheets can be integrated with different device structures as they possess some potential physical and chemical properties and this attracted great interest among researchers to apply them for preparing wearable and portable energy storage devices.

Overall, further improvement in metal oxide nanosheets will have a potential role in the significant development of EESs in the upcoming years.

## 7. Conclusions

This article reviews and discusses metal oxide nanosheets and their suitable applications in several electrochemical energy storage devices. With the rising energy demand, the requirement for advancements in energy generation and energy storage materials is also increasing and these necessities inspire researchers to carry out effective and potential research. This work reviews several MO synthesis processes that are based on top-down and bottom-up processes, as well as their advancements over time. Researchers are primarily focused on preparing MO nanosheets in a pathway that can improve power and energy density in supercapacitors, retention ability in batteries, and surface area and electrocatalytic performance in fuel cells. This paper reviews extensively the applicability, and variation of the performance of several MO nanosheets in different operating conditions for various applications, such as the battery, supercapacitors, and fuel cells. For further study and improvement of MO nanosheets, this article gives detailed information and guidelines from which researchers can obtain a lot of information on MO nanosheets and their applicability and performance parameters in different electrochemical energy storage devices.

## Figures and Tables

**Table 1:** TBAOH-based nanosheets and their applications in energy storage devices.

Nanosheet Compound	Approach	Energy Storage Application	Ref.
RuO_2_	Acid-based TBAOH [41]	Fuel cell, batteries, supercapacitor	[50,51]
Ti_4_O_9_	Acid-based TBAOH [52]	Fuel cells, batteries	[53,54]
Fe_0.8_Ti_1.2_O_4_	Acid-based TBAOH [46]	Batteries	[55]
Ti_0.87_O_2_	Acid-based TBAOH [43]	Batteries, supercapacitors	[56,57]
Ti_0.91_O_2_	Acid-based TBAOH [42,58]	Fuel cells, batteries	[59]
Mn_1 − x_Ru_x_O_2_ (x = 0.1 and 0.05)	Acid-based TBAOH [60]	Supercapacitors	[60]
MnO_2_	Acid-based TBAOH [61]	Batteries, supercapacitors	[62,63]

**Table 2:** Methodologies for nanosheet synthesis with their advantages and limitation.

Method	Examples of Materials	Advantages	Limitations
Liquid-metal-based mechanical exfoliation	BiO_2_, Gd_2_O_3_, SnO, HfO_2,_ etc.	1. Large size up to millimeter; 2. Atomically thin 2D-metal oxides; 3. Low-cost; 4. Can be used for individuals whose bulk hosts do not exist.	1. Metal-in-metal solubility dependent; 2. Low yield; 3. Thermodynamic regulation could restrict the compatibility of numerous materials; 4. Fundamentally needs to be explored.
Electrophoretic synthesis	Transitional MO, such as ZnO	1. High production; 2. Simple process; 3. High purity nanosheets with a level of quality comparable to that produced through chemical vapor deposition (CVD).	1. May require corrosive electrolytes. 2. Not all yielding factors have been analyzed; 3. Not suitable for some types of materials.
Hydrothermal/ solvothermal synthesis	Co_3_O_4_, ZnO, NiO_2_, Fe_3_O_4_, TiO_2_ etc.	1. High specific surface area; 2. Flexible and controlled working conditions; 3. Gram-scale productions; 4. Compatibility and scalability with flexible substrates; 5. Applicable to any material.	1. Generally, produces harmful side products; 2. Little power over dimensions and mechanism.
Graphene assisted synthesis	Al_2_O_3_, Cu_2_O, MnO_2_ etc.	1. Highly crumpled and wrinkled structures; 2. Large surface area.	1. Only suitable for a few applications; 2. Depends on the characteristics of graphene; 3. Graphene removal challenges; 4. May bring carbonaceous impurities.
Liquid exfoliation	V_2_O_5_, Co_3_O_4_, MoO_3_ etc.	1. Exfoliation is typically performed using low-boiling liquids; 2. Relatively simple approach; 3. Can be high yielding; 4. Does not necessitate any advanced equipment.	1. Poor control over layer thickness; 2. Introduction of unwanted defects; 3. Little control over lateral dimensions; 4. Inapplicable to components for which bulk host does not persist.
Chemical vapor deposition	TiO_2_, V_2_O_5_, MoO_3_	1. Effective regulation of expansion; 2. Management of size and thickness; 3. Compatible with an exquisite coating over functional material.	1. Has a lengthy processing duration and necessitates high temperatures; 2. Produces limited output; 3. Expensive and only suitable for advanced applications; 4. High energy consumption; 5. The quality can be reduced by irregular nucleation.
Ion-intercalation	W_2_O_7_, K_0.45_MnO_2_, TaO_3_, RuO_2_ etc.	1. Low energy requirement; 2. High-production; 3. High-precision in controlling the thickness of single-layer sheets.	1. Needs severe chemical environments; 2. Bulk host influences badly the 2D structure; 3. Cannot be used for substances without having a host.
Salt-assisted growth	K_0.27_MnO_2_·0.54H_2_O, Li_2_WO_4_, etc.	1. Fine doping of heteroatoms; 2. Low-cost; 3. High-purity; 4. Applicable to	1. A relatively high temperature is necessary; 2. Unwanted heteroatoms from salt can cause contamination through doping; 3. There are still many uncertainties regarding the fundamental aspects of the process.
Liquid-metal-based mechanical exfoliation	BiO_2_, Gd_2_O_3_, SnO, HfO_2_ etc.	1. Large size up to millimeter. 2. Atomically thin 2D-metal oxides 3. Low-cost. 4. Can be used for individuals whose bulk hosts do not exist.	1. Metal-in-metal solubility dependent 2. Low yield. 3. Thermodynamic regulation could restrict the compatibility of numerous materials. 4. Fundamental needs to be explored.
Electrophoretic synthesis	Transitional MO such as ZnO	1. High production. 2. Simple process. 3. great purity nanosheets with a level of quality comparable to that produced through Chemical Vapor Deposition (CVD).	1. May requisite electrolytes-corrosive. 2. All yielding factors are analyzed yet. 3. Not suitable for all types of materials.
Hydrothermal/ solvothermal synthesis	Co_3_O_4_, ZnO, NiO_2_, Fe_3_O_4_, TiO_2_ etc.	1. High specific surface area. 2. Flexible and controlled working conditions. 3. Gram-scale productions. 4. Compatibility and scalability with flexible substrates. 5. Applicable to any material.	1. Generally, produces harmful side products. 2. small power over dimensional and mechanism.
Graphene assisted synthesis	Al_2_O_3_, Cu_2_O, MnO_2_ etc.	1. Highly crumpled and wrinkled structures. 2. Large surface area.	1. Only suitable for a few applications. 2. Depend on the characteristics of graphene. 3. Graphene removal challenges. 4. May bring carbonaceous impurities.
Liquid exfoliation	V_2_O_5_, Co_3_O_4_, MoO_3_ etc.	1. Exfoliation is typically performed using low-boiling liquids. 2. Relatively simple approach. 3. Can be high yield 4. Does not necessitate any advanced equipment.	1. Poor control over layer thickness. 2. Introduction of unwanted defects. 3. small control over lateral dimensions. 4. Inapplicable to components for which bulk host does not persist.
Chemical vapor deposition	TiO_2_, V_2_O_5_, MoO_3_	1. Effective regulation of expansion 2. Management of size and thickness. 3. Compatible with an exquisite coating over functional material.	1. Has a lengthy processing duration and necessitates high temperatures. 2. Produces limited output. 3. Expensive and only suitable for advanced applications. 4. High energy consumption. 5. The quality can be reduced by irregular nucleation.
Ion-intercalation	W_2_O_7_, K_0.45_MnO_2_, TaO_3_, RuO_2_ etc.	1. Low energy requirement. 2. High-production. 3. High-precision in controlling the thickness of single-layer sheets.	1. Needs severe chemical environments. 2. Bulk host influences badly the 2D structure 3. Can’t be used for substances without having a host.
Salt-assisted growth	K_0.27_MnO_2_·0.54H_2_O, Li_2_WO_4_, etc.	1. Fine doping of heteroatoms. 2. Low-cost. 3. High-purity. 4. Applicable to	1. A relatively high temperature is necessary. 2. Unwanted heteroatoms from salt can cause contamination through doping. 3. There are still many uncertainties regarding the fundamental aspects of the process.

**Table 3:** Battery-supercapacitor hybrid (BSH) systems with their performance parameters.

BSH Structure	Electrolyte	Performance Parameters	Power Density	Energy Density	Ref.
Na-ion Batteries					
Activated Carbon//NaMnO_2_	Aqueous Na_2_SO_4_ solution	97% after 10,000 cycles, 10 C	13.2 Wh kg^−1^ at 1 kW kg^−1^	19.5 Wh kg^−1^ at 130 kW kg^−1^	[112]
AC//V_2_O_5_-CNT	Organic NaClO_4_	80% after 900 cycles, 60 C	7.5 Wh kg^−1^ at 5000 W kg^−1^	38 Wh kg^−1^ at 140 W kg^−1^	[113]
AC//Na_4_Mn_9_O_18_	Aqueous Na_2_SO_4_ solution	84% after 4000 cycles	21 Wh kg^−1^ at 337.4 W kg^−1^	34.8 Wh kg^−1^ at 62 W kg^−1^	[114]
CoHCF//carbon microspheres	Aqueous Na_2_SO_4_ solution	92% after 1000 cycles	37.8 Wh kg^−1^ at 5037 W kg^−1^	54.4 Wh kg^−1^ 800 W kg^−1^	[115]
Li-ion batteries					
LiNi_0.5_Mn_1.5_O_4_//AC	LiPF_6_	89% after 4000 cycles, 10 C	63 Wh kg^−1^ at 100,100 W kg^−1^	40 Wh kg^−1^ at 1000 W kg^−1^	[116]
AC//LiNi_0.5_Mn_1.5_O_4_	LiPF_6_	81 % after 3000 cycles	8 Wh kg^−1^ at 2.5 kW kg^−1^	19 Wh kg^−1^ at 150 W kg^−1^	[117]
CNTs//Li_4_Ti_5_O_12_	LiPF_6_	92% after 3000 cycles			[50]
Fe_3_O_4_/G//3D graphene	LiPF_6_	70% after 1000 cycles,	86 Wh kg^−1^ at 2587 W kg^−1^	147 Wh kg^−1^ at 150 W kg^−1^	[118]
N-doped graphene//Li_4_Ti_5_O_12_	LiPF_6_	64 after 10,000 cycles	21 Wh kg^−1^ at 8000 W kg^−1^	70 Wh kg^−1^ at 200 W kg^−1^	[119]
Acidic BSH					
PbO_2_//AC	Aqueous H_2_SO_4_ solution	80% after 3000 cycles, 10 C	18 Wh kg^−1^ at 691 W kg^−1^	27 Wh kg^−1^ at 152 W kg^−1^	[120]
		835 after 3000 cycles, 4 C	17.8 Wh kg^−1^ at 500 W kg^−1^	26.5 Wh kg^−1^ at 30.8 W kg^−1^	[121]
C/PbO_2_	0.1 M CH_3_SO_3_H/ Pb(NO_3_)_2_ +4M NaNO_3_	100% after 5000 cycles, 22C	-	29 Wh kg^−1^	[122]
NiMoO_4_//AC	Aqueous KOH	14.3% after 10000 cycles	41.1 Wh kg^−1^ at 17,002 W kg^−1^	60.9 Wh kg^−1^ at 850 W kg^−1^	[123]
CNTs//Fe_3_O_4_-C	Aqueous KOH	67.6% AFTER 1000 cycles	-	1.56 mWh cm^−3^ at 0.028 W cm^−3^	[124]

**Table 4:** Applications of several MO nanosheets in different EES systems.

Energy Storage Type	MO Nanosheet	Performance	Ref.
Li-ion batteries	superlattice	370 mAh g^−1^ (12.8 A g^−1^)	[160]
		480 mA h^−1^ (5 A g^−1^, 5000 cycles)	
	MnO_2_/graphene	1325 mAh g^−1^ (0.1 A g^−1^)	
	Mg, Li, Na, Co, K-MnO_2_	85–160 mAh g^−1^ (30 mA g^−1^)	[161]
	MnO_2_/MoS_2_	700 mAh g^−1^ (200 mA g^−1^)	[162]
	Li-MnO_2_	266 mAh g^−1^ (44 μA cm^−2^)	[163]
	V_2_O_5_	141 mAh g^−1^ (100 mA g^−1^)	[164]
	MnO/rGO	1607 mAh g^−1^ (100 mA g^−1^)	[165]
	FeO/rGO	1412 mAh g^−1^ (100 mA g^−1^)	
	Li-Mn_3_O_4_	250 mA h g^−1^ (600 mAg^−1^)	[166]
Li-S batteries	MnO_2_/ carbon nanofibers	1156 mAh g^−1^ (0.2 C)	[167]
		856.1 mAh g^−1^ (0.5 C, 200 cycles)	
	S/Ti_0.87_O_2_ cathode (with 80 wt% sulfur content)	82.3% (1000 mA g^−1^, 300 cycles)	[78]
		1023.5 mAh g^−1^ (50 mA g^−1^)	
	S/MnO_2_ cathode (75 wt% sulfur content)	245 mAh g^−1^ (2 C, 2000 cycles)	[168]
		1300 mAh g^−1^ (0.05 C)	
Na-ion batteries	MnO_2_/graphene superlattice	245 mAh g^−1^ (12.8 A g^−1^)	[169]
		185 mAh g^−1^ (5 Ag^−1^, 5000 cycles)	
		795 mAh g^−1^ (0.1 A g^−1^)	
	Na, K, Li, Co, Mg-MnO_2_	45–145 mAh g^−1^ (30 mA g^−1^)	[161]
	FeO_X_	263.4 mAh g^−1^ (100 mA g^−1^)	[106]
Zn-ion batteries	K-δ-MnO_2_	270.5 mAh g^−1^ (1000 cycles, 0.1 A g^−1^)	[170]
		95.1 mAh g^−1^ (1000 cycles, 3 A g^−1^)	
	K_0.19_MnO_2_·0.56 H_2_O	107 mAh g^−1^ (~87.5% capacity retention after 2000 cycle)	[171]
	MnCo_2_O_4_	848 mAh g^−1^ (200 cycles, 0.2 A g^−1^)	[172]
	S-MnO_2_	324 mAh g^−1^ (200 mA g^−1^)	[173]
		205 mAh g^−1^ (2000 mA g^−1^)	
	P-Co_3_O_4_	244.9 mAh g^−1^ (at 4 A g^−1^)	[174]
	P-Co_3_O_4_/Zn	119.4 mAh g^−1^ (5000 cycles, 1 A g^−1^)	
Zn-air batteries	N/P-Cu_0.1_Co_0.3_Mn_0.6_O_2_/CNT	108.1 mW cm^−2^ (200 h at 10 mA cm^−2^)	[175]
	Fe-Mn_3_O_4_	740 mAh g^−1^ (108 h, 5 mA cm^−2^)	[176]
Supercapacitors	MnO_2_/Ti_3_C_2_	192 F g^−1^ (80 mV s^−1^) 346 F g^−1^ (10 mV s^−1^)	[156]
		240 F g^−1^ (100 mV s^−1^) 390 F g^−1^ (10 mV s^−1^) 93% (1 A g^−1^, 6000 cycles)	[177]
	rGO/MXene@NiCoO_2_	1614 F g^−1^ (0.5 A g^−1^) 1257.5 F g^−1^ (10 A g^−1^)	[178]
	Freeze-dried MnO_2_	110 F g^−1^ (0.2 A g^−1^) 160 F g^−1^ (0.2 A g^−1^) 80–100% (5000 cycles, 5 A g^−1^)	[154]
	Co_3_O_4_ nanoparticles-MXene (Co-MXene)	1081 F g^−1^ (0.5 A g^−1^)	[179]
	MnO_2_	868 F g^−1^ (3 A g^−1^) 91% (10,000 cycles, 3 A g^−1^)	[155]
	RuO_2_	700 F g^−1^ (2 mV s^−1^)	[153]
	M-NC@NCM/NF	2137.5 F g^−1^ (1 A g^−1^)	[180]
	Bi_2_MoO_6_	655.5 mF cm^−2^ (1 mA cm^−2^)	[181]
	CuCo_2_O_4_	1210 F g^−1^ (2 A g^−1^)	[182]
	CuCo_2_O_4_@NiO	124.6 F g^−1^ (1 A g^−1^) ∼81.3%, 6000 cycles	[183]
	CuCo_2_O_4_/NiO	2219 F g^−1^ (1 A g^−1^) ∼95.3% after 10,000 cycles 1405 F g^−1^ (20 A g^−1^)	[184]
	CuCo_2_O_4_ nanosheets@MnO_2_	416 F g^−1^ (1 A g^−1^) (92.1% retention after 4200 cycles)	[185]
	ZnNiCo-P	~ 958 C g^−1^ (1 A g^−1^)	[186]
	2D MnO_2_	356.11 F g^−1^ (5 mV s^−1^)	[34]
	MnO_2_	543 F g^−1^ at scan rate 5 mV s^−1^	[70]
	Mn_3_O_4_	127 F g^−1^ (0.5 A g^−1^)	[71]
	MnO_2_	87.1 F g^−1^ (95.2 % retention after 3000 Cycles)	[158]
Solid oxide fuel cell	La_0.6_Sr_0.4_CoO _3 − δ_	1.2 W cm^−2^ at 600 °C	[133]
Alkaline fuel cell	MF assisted QPVA/0.1%Fe_3_O_4_@GO	200 mW cm^−2^	[187]
Microbial fuel cell	77% NiO/CNT	670 mW cm^−2^	[149]
	TiO_2_-20PANI/CP	813 mW cm^−2^	[148]
PEM fuel cell	Pt-NiO_2 − x_NS	958 mW cm^−2^	[140]

## Data Availability

No new data were created or analyzed in this study. Data sharing does not apply to this article.
